# Potassium Homeostasis and the Systemic Consequences of Intracellular Potassium Deficiency: From Molecular Mechanisms to Clinical Manifestations

**DOI:** 10.3390/biom16081127

**Published:** 2026-08-03

**Authors:** Vladimir Ivashkin, Oksana Zolnikova, Victoria Agarkova, Svetlana Appolonova, Vadim Tarasov, Roman Maslennikov, Elena Poluektova, Konstantin Ivashkin

**Affiliations:** 1 Department of Internal Medicine, Gastroenterology and Hepatology, Sechenov University, Moscow 119435, Russia; zolnikova_o_yu@staff.sechenov.ru (O.Z.); agarkova_v_yu@staff.sechenov.ru (V.A.); appolonova_s_a@staff.sechenov.ru (S.A.); maslennikov_r_v@staff.sechenov.ru (R.M.); poluektova_e_a@staff.sechenov.ru (E.P.); ivashkin_k_v_1@staff.sechenov.ru (K.I.); 2 Scientific and Technological Institute of Metabolic Health, Sechenov University, Moscow 119435, Russia; 3 Institute of Translational Medicine and Biotechnology, Sechenov University, Moscow 119435, Russia; tarasov_v_v_2@staff.sechenov.ru

**Keywords:** potassium homeostasis, hypokalemia, intracellular potassium deficiency, potassium depletion, Na^+^/K^+^-ATPase, mitochondrial dysfunction, insulin resistance, metabolic alkalosis, inflammation, anabolic resistance

## Abstract

Potassium is the predominant intracellular cation and plays a fundamental role in maintaining membrane potential, cellular metabolism, enzyme activity, protein synthesis, acid–base homeostasis, and mitochondrial bioenergetics. Although hypokalemia is readily identified by decreased serum potassium concentration, serum potassium represents only a small fraction of total-body potassium stores and therefore may not accurately reflect intracellular potassium status. Consequently, chronic total-body potassium depletion and intracellular potassium deficiency may remain clinically unrecognized despite serum potassium concentrations within the reference range. Growing experimental and clinical evidence suggests that disturbances of potassium homeostasis are associated with multiple metabolic abnormalities involving the cardiovascular, neuromuscular, endocrine, renal, and gastrointestinal systems. Intracellular potassium depletion has been associated with mitochondrial dysfunction, oxidative stress, impaired insulin secretion and insulin sensitivity, metabolic alkalosis, chronic low-grade inflammation, and anabolic resistance. However, these relationships are complex and frequently bidirectional, indicating that potassium deficiency should be regarded as one component of an integrated metabolic network rather than an isolated pathogenic mechanism. This narrative review critically summarizes current evidence regarding the physiological regulation of potassium homeostasis, distinguishes hypokalemia from total-body potassium depletion and intracellular potassium deficiency, and discusses the mechanisms through which potassium depletion may contribute to systemic metabolic dysfunction. Particular attention is given to the clinical manifestations, diagnostic evaluation, and current therapeutic approaches to disorders of potassium homeostasis. Finally, important knowledge gaps are highlighted, including the need for reliable biomarkers of intracellular potassium deficiency and prospective studies evaluating whether correction of chronic potassium depletion improves long-term metabolic and clinical outcomes beyond normalization of serum potassium concentration.

## 1. Introduction

Potassium is the predominant intracellular cation and an essential determinant of cellular excitability, membrane potential, osmotic balance, enzyme activity, and energy metabolism. Approximately 98% of total body potassium resides within the intracellular compartment, whereas only about 2% is present in the extracellular fluid, where serum potassium concentration is tightly regulated within a narrow physiological range [1,2,3,4,5,6,7,8,9]. Maintenance of this transmembrane gradient depends primarily on coordinated Na^+^/K^+^-ATPase activity, renal potassium handling, hormonal regulation, and transcellular potassium redistribution [1,4,6,7,10].

Several closely related but distinct concepts are frequently used interchangeably in both clinical practice and the scientific literature. Hypokalemia refers exclusively to a decrease in serum potassium concentration below the lower limit of the reference range. Total-body potassium depletion denotes a reduction in whole-body potassium stores regardless of serum potassium concentration. Intracellular potassium deficiency describes reduced potassium content within cells and tissues and may occur with either normal or decreased serum potassium levels depending on the underlying pathophysiological mechanisms [4,7,10,11,12,13]. Because no validated clinical method currently exists for direct measurement of intracellular potassium stores, this condition is inferred from the clinical context, potassium balance, urinary potassium excretion, associated electrolyte abnormalities, and the patient’s response to potassium replacement rather than measured directly [4,7,11,13].

Hypokalemia may result from true potassium loss through the kidneys or gastrointestinal tract, inadequate dietary intake, or redistribution of potassium into the intracellular compartment. Conversely, patients with chronic potassium depletion may maintain normal serum potassium concentrations because of adaptive renal conservation and redistribution between extracellular and intracellular compartments. Therefore, serum potassium concentration alone should not be considered a reliable surrogate marker of total-body potassium stores or intracellular potassium content [4,7,13].

Although these conditions frequently coexist, they should not be regarded as synonymous. Accordingly, the broader term disturbances of potassium homeostasis is used throughout this review whenever the available evidence does not permit clear distinction between serum hypokalemia, intracellular potassium depletion, and total-body potassium depletion.

Increasing experimental and clinical evidence demonstrates that disturbances of potassium homeostasis are associated with impaired glucose metabolism, mitochondrial dysfunction, acid–base disturbances, cardiovascular disease, neuromuscular dysfunction, and gastrointestinal dysmotility. In most clinical settings, however, these relationships are bidirectional, with potassium imbalance acting both as a consequence of the underlying disease and as a factor capable of amplifying metabolic dysfunction [2,4,10,11,13,14].

The present review focuses primarily on intracellular potassium deficiency and chronic total-body potassium depletion while discussing their relationship with serum hypokalemia whenever clinically relevant. Particular emphasis is placed on the physiological mechanisms regulating intracellular potassium homeostasis, the systemic metabolic consequences of potassium depletion, and its potential contribution to cardiometabolic, neurological, and gastrointestinal disorders.

## 2. Methods

This article was prepared as a narrative review of the current literature concerning potassium homeostasis and intracellular potassium deficiency.

Literature searches were conducted using PubMed/MEDLINE, Scopus, and Google Scholar for publications available through January 2026. Search terms included combinations of potassium homeostasis, hypokalemia, potassium depletion, intracellular potassium, Na^+^/K^+^-ATPase, potassium metabolism, mitochondrial dysfunction, insulin resistance, metabolic alkalosis, and gastrointestinal motility.

Original research articles, systematic reviews, meta-analyses, clinical practice guidelines, and landmark experimental studies were considered. Greater emphasis was placed on contemporary human studies and international clinical guidelines, whereas earlier publications were included when they established fundamental physiological concepts that remain relevant to the current understanding of potassium homeostasis [4,7,11,13,15].

Because direct assessment of intracellular potassium stores is not routinely available in clinical practice and the available evidence remains heterogeneous, this review was designed as a narrative rather than a systematic review.

## 3. Potassium Metabolism in Humans

Potassium (K^+^) is not produced by the human body but is obtained exclusively through diet. Its main natural sources are fresh and unprocessed foods. Many fruits and vegetables, legumes (such as soybeans), potatoes, meat, poultry, fish, milk, yogurt, and nuts are rich in K^+^. Among starchy products, whole-grain flour and brown rice contain much more K^+^ than their refined counterparts (white wheat flour and white rice).

**Figure 1:**
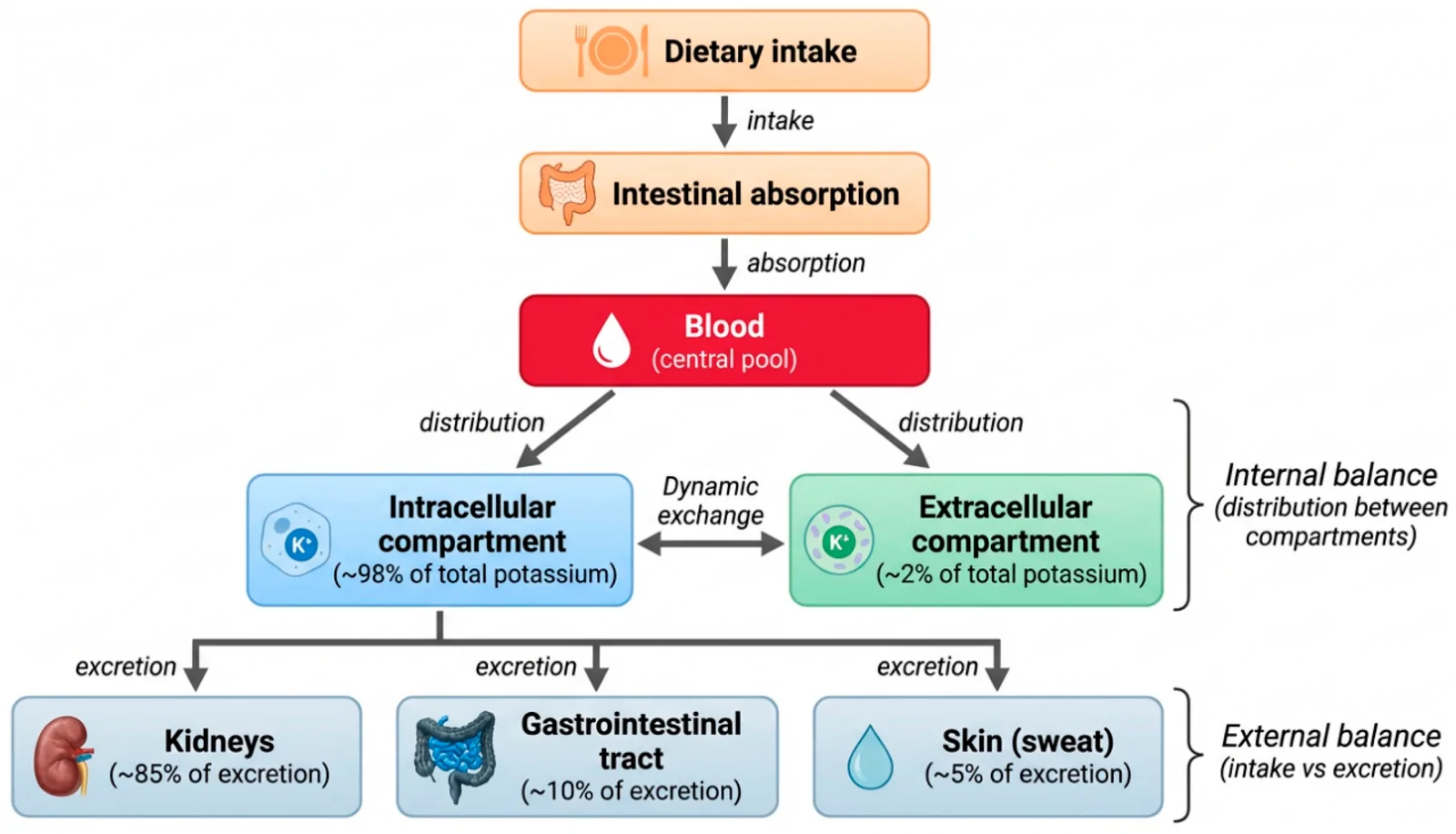
Scheme of Potassium Metabolism in the Human Body.

K^+^ is almost completely absorbed (90–95%) in the small intestine via passive diffusion [2,4]. Homeostasis of K^+^ in the organism is primarily maintained through renal excretion in the distal nephrons rather than restricted absorption, regulated by aldosterone [2,3,4]. In response to increased plasma levels or total body content of K^+^, aldosterone secretion increases, enhancing urinary excretion of K^+^. This mechanism has high speed and effectiveness, allowing the body to adapt to significant fluctuations in dietary K^+^ intake. Thus, kidneys are the primary route of elimination of K^+^ (~85%); approximately 10% is eliminated through the gastrointestinal tract, with only a minor fraction (~5%) accounted for by sweating (Figure 1) [1,4,5].

Potassium (K^+^) is essential for the proper functioning of all organs and systems [3]. It should be noted that even in conditions of complete absence of external intake of potassium, the body continues to lose it through so-called obligatory pathways—via urine, feces, and sweat. For example, minimal renal excretion of potassium amounts to about 5 mmol/day (approximately 195 mg), losses via feces vary between 5 and 10 mmol/day, while skin loss (through sweating) averages at approximately 1–2 mmol/day. However, this last figure can significantly increase under hyperthermic or intense physical activity conditions [6,7]. The total inevitable losses determine the minimum physiological requirement for potassium. To maintain a neutral balance (where intake equals output), an intake of no less than 500–900 mg (10–20 mmol) of potassium per day is necessary. According to modern concepts, however, optimal consumption levels are much higher, amounting to 3.5–4.7 g/day [2,3,4].

The World Health Organization (WHO) conditionally recommends a potassium intake of at least 90 mmol/day, which equates to approximately 3510 mg/day for adults (aged 16 and older) of potassium (K^+^) and less than 2 g of sodium (Na^+^) daily (equivalent to roughly 5 g of table salt) [3]. Nevertheless, these recommendations are practically never met anywhere globally. A recent systematic review and meta-analysis of 104 studies from 52 countries found that none of the studied countries’ average intakes of potassium and sodium align with recommended norms [2]. Moreover, there are pronounced regional differences. For instance, Asian countries exhibit the lowest potassium intake (average consumption of 1.89 g/day) concurrently with the highest sodium intake. Eastern and Western European nations show somewhat higher potassium consumption closer to guidelines (with 31% consuming more than 2.5 g of potassium daily and 14% over 3.5 g/day), but still retain excessive sodium intake [2].

## 4. Physiological Role of Potassium

Maintenance of intracellular potassium concentration is essential for normal cellular function. Beyond its fundamental role in establishing the resting membrane potential, potassium participates in the regulation of enzyme activity, protein synthesis, cellular metabolism, mitochondrial bioenergetics, intracellular signaling, and acid–base homeostasis. Because virtually every cell depends on an adequate intracellular potassium concentration, disturbances of potassium homeostasis may affect nearly every organ system. Skeletal muscle, myocardium, nervous tissue, kidneys, and the gastrointestinal tract are particularly vulnerable because of their high metabolic activity and substantial intracellular potassium content [5,13].

One of potassium’s principal physiological functions is maintenance of the resting membrane potential. The steep transmembrane potassium gradient generated by Na^+^/K^+^-ATPase provides the electrical basis for excitability in neurons, skeletal muscle, cardiac myocytes, and smooth muscle cells. Even relatively small changes in extracellular potassium concentration alter membrane polarization and therefore influence neuromuscular transmission, cardiac impulse conduction, vascular tone, and gastrointestinal motility. Clinically, these alterations may manifest as muscle weakness, cardiac arrhythmias, impaired intestinal motility, or neurological dysfunction [11,13].

Intracellular potassium also serves as an essential cofactor for numerous enzymes involved in carbohydrate metabolism, nucleic acid synthesis, protein synthesis, and cellular growth. Experimental studies have consistently demonstrated that potassium depletion suppresses protein synthesis, enhances protein degradation, and alters intracellular metabolic pathways. Although these metabolic changes become particularly evident during chronic potassium depletion, they are usually amplified by concomitant inflammation, endocrine disorders, reduced physical activity, and malnutrition rather than resulting from potassium deficiency alone [13,14].

Potassium homeostasis is closely linked to mitochondrial function. Potassium fluxes across the inner mitochondrial membrane contribute to regulation of mitochondrial matrix volume, maintenance of membrane potential, oxidative phosphorylation, ATP synthesis, and reactive oxygen species (ROS) production. Disturbances of intracellular potassium balance therefore have the potential to impair mitochondrial bioenergetics. However, available evidence suggests that mitochondrial dysfunction associated with potassium depletion develops through multiple interacting mechanisms involving oxidative stress, inflammation, neurohormonal activation, and the underlying disease process rather than through a single direct effect of potassium deficiency [13,16,17].

Potassium also plays an important role in acid–base regulation. Alterations in extracellular potassium concentration influence hydrogen ion transport across cell membranes and modify renal ammoniagenesis, bicarbonate reabsorption, and distal tubular acid secretion. Consequently, potassium depletion and metabolic alkalosis frequently coexist, although their relationship is reciprocal and depends on the underlying clinical disorder [13,18,19].

Increasing evidence further indicates that intracellular potassium participates in the regulation of inflammatory signaling. Experimental studies have shown that a decrease in intracellular potassium concentration facilitates activation of the NLRP3 inflammasome, thereby promoting maturation of interleukin-1β and interleukin-18. Nevertheless, inflammasome activation represents only one component of a highly complex inflammatory network, and its clinical significance varies according to the underlying pathological condition. Accordingly, potassium depletion should be regarded as a modulator of inflammatory responses rather than their universal initiating factor [13,14,20,21].

Recent studies have also demonstrated important interactions between potassium homeostasis and glucose metabolism. Potassium deficiency may impair insulin secretion, reduce insulin sensitivity, and alter glucose utilization within skeletal muscle and other peripheral tissues. These relationships are influenced by obesity, activation of the renin–angiotensin–aldosterone system, chronic kidney disease, systemic inflammation, and other metabolic abnormalities. Consequently, available evidence supports an association between potassium depletion and impaired glucose metabolism rather than a simple causal relationship [14,22,23,24,25,26,27,28,29,30].

Collectively, these diverse physiological functions explain why disturbances of potassium homeostasis produce multisystem clinical manifestations (Table 1).

## 5. Potassium Deficiency Syndrome

Disturbances of potassium homeostasis frequently develop gradually over weeks or months and may remain clinically unrecognized until substantial depletion of intracellular and total-body potassium stores has occurred. Because serum potassium reflects only a small fraction of total-body potassium, chronic potassium depletion may exist despite serum potassium concentrations within the reference range. Consequently, the clinical consequences of potassium deficiency are determined not only by serum potassium concentration but also by the magnitude, duration, and underlying mechanism of potassium imbalance [4,7,11,13,14].

The concept of potassium deficiency syndrome, first proposed by V. T. Ivashkin in the early 1980s, describes the multisystem functional disturbances associated with chronic potassium depletion [5]. Although this term is not used in contemporary international clinical practice, it remains valuable as a conceptual framework for understanding the systemic metabolic consequences of prolonged intracellular potassium deficiency. Throughout the present review, the term is used primarily to refer to chronic intracellular potassium depletion and total-body potassium deficiency.

Experimental studies demonstrate that chronic potassium depletion induces adaptive cellular responses involving altered Na^+^/K^+^-ATPase activity, changes in transmembrane ion transport, activation of the renin–angiotensin–aldosterone system, increased renal ammoniagenesis, oxidative stress, and remodeling of intracellular metabolic pathways. Initially, these adaptive responses preserve extracellular potassium concentration; however, persistent compensation may occur at the expense of progressive intracellular potassium depletion and functional impairment of metabolically active tissues [13,18,19,31].

Accumulating evidence further indicates that chronic potassium depletion is associated with a broad spectrum of metabolic abnormalities, including oxidative stress, mitochondrial dysfunction, systemic inflammation, insulin resistance, endothelial dysfunction, metabolic alkalosis, and impaired protein synthesis. However, these relationships are highly interconnected and frequently bidirectional. Some of the mechanisms are well characterized and firmly established, whereas others remain incompletely elucidated. Potassium deficiency should therefore be regarded as one component of an integrated pathophysiological network rather than an isolated pathogenic mechanism [13,20,21,22,23,24,25,26,27,28,29,30,31,32,33,34,35].

The following sections discuss the principal mechanisms through which chronic potassium depletion may contribute to systemic metabolic disturbances while distinguishing well-established physiological concepts from mechanisms that remain incompletely understood.

### 5.1. Systemic Inflammation

Chronic low-grade systemic inflammation is increasingly recognized as one of the principal mechanisms contributing to metabolic dysfunction and progression of numerous chronic diseases. Current evidence indicates that disturbances of potassium homeostasis and inflammatory activation are linked through complex bidirectional interactions. Intracellular potassium depletion may amplify inflammatory signaling, whereas persistent inflammation may simultaneously impair potassium homeostasis through multiple complementary mechanisms [13,14].

Among the most extensively investigated mechanisms is activation of the NLRP3 inflammasome. Experimental studies have demonstrated that a reduction in intracellular potassium concentration serves as an important permissive signal for assembly of the NLRP3 inflammasome complex, leading to activation of caspase-1 and subsequent maturation of interleukin-1β and interleukin-18. Nevertheless, potassium efflux alone is insufficient to initiate inflammation. Inflammasome activation requires the coordinated action of pathogen-associated or danger-associated molecular patterns together with additional cellular stress signals. Therefore, intracellular potassium depletion should be regarded as a facilitator rather than the sole trigger of inflammatory activation [20,21].

Inflammation may, in turn, aggravate disturbances of potassium homeostasis through several mechanisms. Pro-inflammatory cytokines, oxidative stress, mitochondrial dysfunction, activation of the sympathetic nervous system, glucocorticoid excess, and stimulation of the renin–angiotensin–aldosterone system may impair Na^+^/K^+^-ATPase activity, alter transmembrane potassium distribution, and modify renal potassium handling. The relative contribution of these mechanisms varies according to the underlying disease and cannot be generalized across all inflammatory conditions [7,13,14].

Oxidative stress represents another important link between inflammation and potassium homeostasis. Excessive generation of reactive oxygen species promotes lipid peroxidation, alters membrane fluidity, and impairs the function of ion channels and membrane transport proteins, including Na^+^/K^+^-ATPase. Progressive impairment of membrane transport further disrupts intracellular potassium distribution, creating a vicious cycle in which oxidative stress and potassium depletion reinforce one another [14,16,17].

Experimental models have further demonstrated that chronic β-adrenergic stimulation, hypercortisolemia, intracellular calcium overload, endocrine disorders, tissue hypoxia, chronic kidney disease, and several commonly prescribed medications may modify intracellular potassium balance both directly and indirectly. Consequently, potassium disturbances observed during chronic inflammatory diseases should be regarded as multifactorial metabolic adaptations rather than the consequence of a single pathogenic mechanism [13,14].

Taken together, current evidence supports the concept that intracellular potassium depletion and systemic inflammation participate in a self-reinforcing metabolic network involving oxidative stress, mitochondrial dysfunction, neurohormonal activation, and immune dysregulation. However, available clinical data do not support the conclusion that potassium deficiency alone is sufficient to initiate chronic inflammation. Rather, disturbances of potassium homeostasis should be considered one component of an integrated metabolic response accompanying chronic disease.

### 5.2. Insulin Resistance and Glucose Metabolism

Disturbances of potassium homeostasis are closely associated with abnormalities of glucose metabolism affecting both insulin secretion and peripheral insulin sensitivity. These relationships are complex and bidirectional. Potassium depletion may impair glucose homeostasis, whereas diabetes mellitus, hyperglycemia, insulin therapy, and associated endocrine disorders can themselves alter potassium distribution and increase the risk of hypokalemia. Accordingly, potassium deficiency should be viewed as one component of the broader metabolic network underlying disorders of glucose regulation rather than an isolated electrolyte disturbance [13,22,23,24,25,30].

Normal insulin secretion depends on tightly regulated potassium fluxes within pancreatic β-cells. Under physiological conditions, glucose metabolism increases the intracellular ATP/ADP ratio, leading to closure of ATP-sensitive potassium (K_ATP) channels, reduced potassium efflux, and subsequent membrane depolarization. This depolarization subsequently opens voltage-gated calcium channels, allowing calcium influx and triggering exocytosis of insulin-containing secretory granules [13,22,23,30].

Experimental and clinical studies indicate that disturbances of potassium homeostasis may alter β-cell electrophysiology and attenuate glucose-stimulated insulin secretion. Both chronic potassium depletion and acute hypokalemia appear capable of reducing insulin release, although the magnitude of this effect depends on the severity and duration of potassium deficiency and on concomitant metabolic abnormalities.

The mechanisms underlying this effect are complex and may involve altered transmembrane potassium gradients, changes in K_ATP channel conductance, or impaired β-cell membrane excitability [22,23,25,30]. Studies consistently demonstrate impaired glucose tolerance in patients with hypokalemia, particularly during chronic diuretic therapy or primary aldosteronism, where correction of potassium deficiency frequently improves glucose metabolism [22,23,25,30].

Several complementary mechanisms have been proposed to explain the relationship between intracellular potassium depletion and reduced insulin sensitivity. Experimental evidence suggests that potassium deficiency impairs skeletal muscle glucose utilization through alterations in intracellular enzyme activity, mitochondrial ATP production, and insulin signaling pathways. Based on extrapolation from related experimental models, potassium deficiency has been suggested to reduce IRS-1 phosphorylation, impair PI3K signaling, and decrease GLUT4 translocation [14,22,24,25]. However, direct evidence specifically attributing this complete molecular cascade to potassium depletion remains limited, and these putative mechanisms should be viewed as potential contributing pathways rather than established causal effects in vivo.

Nevertheless, available clinical evidence indicates that potassium depletion alone is unlikely to represent an independent cause of insulin resistance. Instead, these molecular abnormalities interact with obesity, chronic inflammation, activation of the renin–angiotensin–aldosterone system, chronic kidney disease, aging, and physical inactivity to determine the overall degree of insulin resistance.

In addition to possible alterations in insulin signaling, experimental studies suggest that potassium depletion may influence glycogen synthesis, oxidative glucose metabolism, and skeletal muscle bioenergetics. Mitochondrial dysfunction and oxidative stress have also been proposed as potential mediators, although direct evidence supporting these mechanisms in humans remains limited [13,25,31].

Although correction of potassium deficiency has been shown to improve glucose metabolism in selected patient populations, particularly individuals receiving thiazide or loop diuretics and patients with primary aldosteronism, current evidence does not support potassium supplementation as an independent strategy for prevention or treatment of insulin resistance in the general population. Additional prospective studies are required to determine the independent metabolic effects of chronic intracellular potassium depletion [15,36,37,38].

### 5.3. Mitochondrial Dysfunction

Mitochondria are the principal organelles responsible for ATP production through oxidative phosphorylation and therefore play a central role in cellular energy metabolism. Normal mitochondrial function depends on tightly regulated ionic homeostasis, including physiological intracellular potassium concentrations. Although potassium is not directly involved in ATP synthesis, potassium fluxes across mitochondrial membranes regulate matrix volume, membrane potential, respiratory chain activity, calcium homeostasis, and reactive oxygen species (ROS) production [13,16].

Experimental evidence suggests that chronic intracellular potassium depletion may impair mitochondrial function through disturbances in ionic homeostasis, reduced Na^+^/K^+^-ATPase activity, altered mitochondrial membrane potential, increased oxidative stress, and decreased efficiency of oxidative phosphorylation [13,16]. Rather than representing a single pathogenic pathway, mitochondrial dysfunction likely results from the combined effects of potassium depletion, inflammation, neurohormonal activation, tissue hypoxia, and the underlying disease process. Reduced ATP production compromises numerous energy-dependent cellular processes, including active ion transport, protein synthesis, muscle contraction, cellular repair, and maintenance of membrane integrity. Concurrently, excessive ROS generation promotes oxidative damage to lipids, proteins, and mitochondrial DNA, further impairing mitochondrial function and amplifying inflammatory signaling [16,17].

The interaction between potassium homeostasis and mitochondrial metabolism is particularly evident in skeletal muscle. Experimental studies indicate that intracellular potassium depletion may impair mitochondrial substrate utilization, suppress pyruvate oxidation, reduce Krebs cycle activity and fatty acid β-oxidation, and decrease respiratory efficiency. These metabolic abnormalities contribute to reduced exercise tolerance, early muscle fatigue, and diminished physical performance. However, their clinical expression is strongly influenced by coexisting conditions such as diabetes mellitus, obesity, chronic kidney disease, endocrine disorders, malnutrition, chronic inflammation, and physical inactivity [16,17,25,31].

Recent studies have identified mitochondrial potassium channels as important regulators of mitochondrial permeability transition, calcium handling, and cellular resistance to oxidative stress. Although these channels have attracted considerable interest as potential therapeutic targets, their clinical significance in chronic potassium depletion remains incompletely understood [12].

Morphological changes observed during prolonged potassium depletion are largely secondary to persistent metabolic dysfunction. Early alterations—including reversible cellular swelling, glycogen depletion, intracellular protein and lipid accumulation—reflect chronic impairment of cellular energetics. In severe or prolonged potassium depletion, particularly when accompanied by ischemia, hypoxia, toxic injury, or chronic inflammation, these adaptive changes may progress to irreversible structural damage, fibrosis, and organ dysfunction [13,16].

### 5.4. Metabolic Alkalosis

The relationship between potassium depletion and metabolic alkalosis is reciprocal and clinically important. Chronic potassium depletion promotes both the development and maintenance of metabolic alkalosis, whereas metabolic alkalosis enhances renal potassium wasting, thereby aggravating intracellular potassium deficiency. Consequently, these disorders frequently coexist and reinforce one another through complementary physiological mechanisms rather than representing independent abnormalities. One contributing mechanism involves transcellular ion exchange. During potassium depletion, potassium shifts from the intracellular to the extracellular compartment, partially buffering the decline in extracellular potassium concentration. To maintain electroneutrality, hydrogen ions move into cells, producing relative intracellular acidification. However, systemic metabolic alkalosis is sustained predominantly by renal adaptive mechanisms rather than by transcellular ion exchange alone [7,13,18,19].

The kidneys play the central role in maintaining hypokalemic metabolic alkalosis. Potassium depletion enhances distal hydrogen ion secretion, bicarbonate reabsorption, renal ammoniagenesis, and activation of the renin–angiotensin–aldosterone system (RAAS). Aldosterone further promotes sodium reabsorption while increasing urinary potassium and hydrogen ion excretion, perpetuating both hypokalemia and alkalosis. These processes are additionally influenced by distal sodium delivery, chloride availability, extracellular fluid volume, and mineralocorticoid activity [7,13,15,18,19,38].

Clinically, metabolic alkalosis is classified as either chloride-responsive or chloride-resistant based on etiology, volume status, urinary chloride concentration, and mineralocorticoid activity. This distinction is critical for guiding appropriate therapeutic management, as chloride-responsive and chloride-resistant alkalosis require fundamentally different treatment approaches.

Chloride-responsive metabolic alkalosis is typically associated with extracellular volume depletion, gastrointestinal chloride loss (e.g., vomiting, nasogastric suction), previous diuretic therapy, or other conditions accompanied by chloride and volume deficit. In these cases, urinary chloride concentration is typically low (<20–25 mmol/L), and alkalosis generally responds to volume repletion together with chloride and potassium replacement. It is important to emphasize that most cases of hypokalemic metabolic alkalosis encountered in clinical practice belong to this category, as they arise in the setting of diuretic therapy, fluid losses, or gastrointestinal losses [15,38].

Chloride-resistant metabolic alkalosis, by contrast, is most commonly associated with persistent mineralocorticoid excess, including primary aldosteronism, Cushing syndrome, or inherited disorders of mineralocorticoid signaling. In these patients, urinary chloride concentration is typically elevated (>40 mmol/L), and correction of potassium deficiency alone is insufficient without addressing the underlying endocrine disorder; treatment must be directed at the underlying etiology. In clinical practice, chloride-resistant alkalosis is encountered less frequently; however, its recognition is of critical importance, as it may indicate the presence of a serious endocrine disorder [15,38].

Metabolic alkalosis also influences cellular metabolism. Alkalemia shifts the oxyhemoglobin dissociation curve to the left, reducing oxygen unloading to peripheral tissues, and alters intracellular enzyme activity, mitochondrial respiration, and oxidative phosphorylation. However, these metabolic consequences depend largely on the severity and duration of alkalosis and should not be regarded as universal consequences of potassium depletion [7,11,13,38]. Because potassium depletion and metabolic alkalosis perpetuate one another through reciprocal physiological mechanisms, effective management requires simultaneous correction of both abnormalities whenever possible. Identification and treatment of the underlying cause—including gastrointestinal potassium loss, diuretic therapy, renal tubular disorders, mineralocorticoid excess, or chronic kidney disease—are essential to prevent recurrence of both hypokalemia and metabolic alkalosis [7,11,13,38].

### 5.5. Anabolic Resistance

Anabolic resistance refers to the diminished capacity of skeletal muscle and other metabolically active tissues to increase protein synthesis in response to physiological anabolic stimuli such as dietary amino acids, insulin, or physical exercise. Although it is well described in aging, chronic kidney disease, heart failure, cancer, and systemic inflammation, emerging evidence suggests that chronic intracellular potassium deficiency may contribute to this process by altering intracellular metabolic homeostasis [5,18].

Instead, intracellular potassium depletion may induce interconnected disturbances involving mitochondrial dysfunction, impaired ATP generation, changes in intracellular pH, oxidative stress, insulin resistance, and disruption of cellular osmotic balance. Collectively, these changes reduce protein synthesis efficiency while promoting protein degradation. Energy deficiency is a central mechanism. Protein synthesis is highly ATP-dependent, requiring energy for amino acid transport, ribosomal function, peptide biosynthesis, and post-translational modification. As discussed previously, mitochondrial dysfunction associated with potassium depletion reduces ATP availability, thereby limiting energy-dependent protein synthesis [16,17].

Insulin resistance provides an additional link between potassium depletion and impaired protein metabolism. Under physiological conditions, insulin stimulates amino acid uptake, activates the PI3K/Akt/mTORC1 pathway, suppresses proteolysis, and promotes muscle protein synthesis. Chronic potassium depletion may attenuate these anabolic effects indirectly via impaired insulin secretion, reduced insulin sensitivity, and diminished metabolic signaling [22,24,25].

Acid–base disturbances may further contribute. Changes in intracellular pH associated with potassium depletion may affect protein translation and post-translational processing, while alterations in ionic and osmotic balance may impair ribosomal function and protein turnover. Oxidative stress and chronic inflammation further amplify these abnormalities by activating catabolic pathways (ubiquitin–proteasome and autophagy systems) and suppressing anabolic signaling, resulting in a net negative protein balance. Thus, anabolic resistance associated with potassium depletion should be viewed as a multifactorial consequence of impaired bioenergetics, inflammation, endocrine dysregulation, and altered intracellular homeostasis [14,20,21].

Overall, current evidence supports intracellular potassium deficiency as one component of the integrated metabolic network underlying anabolic resistance (Table 2). However, prospective studies are needed to clarify its independent contribution and determine whether correction of intracellular potassium depletion improves long-term functional outcomes.

## 6. Clinical Manifestations

The clinical manifestations of disturbances of potassium homeostasis are highly heterogeneous and depend on the severity, duration, and underlying mechanism of potassium imbalance rather than on serum potassium concentration alone. A 6–10% reduction in total body potassium is not associated with either subjective symptoms or objective clinical manifestations. Functional and morphological changes in internal organs become evident only when the deficit of exchangeable potassium reaches approximately 20–40% of total body potassium stores [5,11]. Acute redistributional hypokalemia may produce prominent neuromuscular and cardiac symptoms despite preserved total-body potassium stores, whereas chronic potassium depletion may remain clinically unrecognized until substantial intracellular potassium deficiency has developed [5,11,12,13].

Neuromuscular manifestations are among the most common clinical features of potassium depletion. Patients may present with generalized fatigue, muscle weakness, reduced exercise tolerance, muscle cramps, paresthesias, and, in severe cases, flaccid paralysis or respiratory muscle involvement. These manifestations primarily reflect impaired membrane excitability resulting from disruption of transmembrane potassium gradients. Chronic intracellular potassium depletion may further contribute to reduced skeletal muscle performance through mitochondrial dysfunction and anabolic resistance [11,13,37].

Cardiovascular manifestations are of particular clinical importance because disturbances of potassium homeostasis significantly increase the risk of cardiac arrhythmias. Typical electrocardiographic abnormalities include flattening or inversion of T waves, ST-segment depression, prominent U waves, QT interval prolongation, and increased ventricular ectopy. Patients with structural heart disease, heart failure, ischemic heart disease, or concomitant use of antiarrhythmic medications are especially susceptible to the proarrhythmic effects of hypokalemia [11,37,38,39,40,41,42,43].

Renal manifestations include impaired urinary concentrating ability, polyuria, polydipsia, nephrogenic diabetes insipidus, enhanced renal ammoniagenesis, and maintenance of metabolic alkalosis through increased distal hydrogen ion secretion and bicarbonate reabsorption. Chronic potassium depletion may also contribute to tubulointerstitial injury and progressive impairment of renal function [13,18,19,43,44,45,46].

Gastrointestinal manifestations result primarily from reduced smooth muscle contractility and include constipation, delayed gastric emptying, abdominal distension, intestinal hypomotility, and, in severe cases, paralytic ileus or intestinal pseudo-obstruction. Patients with chronic gastrointestinal diseases, hereditary tubulopathies, or prolonged diuretic therapy may be particularly vulnerable to these complications [46,47,48]. In addition to its role in gastrointestinal motility, K^+^ is essential for several other physiological processes, including enzyme secretion and activation, epithelial repair, and the maintenance of epithelial barrier integrity [47,48,49,50]. Furthermore, optimal ionic homeostasis is required for the stability of tight junctions. Disruption of electrolyte balance may therefore increase intestinal permeability [47,48,49]. Potassium deficiency has also been proposed to influence the composition of the gut microbiota and to promote the translocation of bacterial lipopolysaccharides, although the available evidence remains limited [51,52,53,54]. Several studies have reported an association between the clinical manifestations of irritable bowel syndrome and reduced levels of various micronutrients, including K^+^ [55,56]. However, to date, there is no evidence supporting a causal relationship or indicating that potassium deficiency represents a primary pathogenic factor in irritable bowel syndrome [55,56]. Rather, hypokalemia is more likely to be a concomitant condition related to dietary restrictions, malabsorption, or chronic stress commonly observed in this patient population. The potential contribution of subclinical potassium deficiency or subtle disturbances in electrolyte homeostasis to the modulation of visceral sensitivity and gastrointestinal motility remains speculative and warrants further well-designed studies.

Neurological and neurovegetative manifestations may include asthenia, decreased concentration, irritability, sleep disturbances, autonomic dysfunction, and nonspecific neurocognitive symptoms. Although these manifestations are not specific for potassium deficiency, they may contribute substantially to reduced quality of life in patients with chronic intracellular potassium depletion [5,11,25,26,27].

Metabolic consequences include impaired glucose tolerance, reduced insulin secretion, and insulin resistance. Experimental evidence further suggests that chronic potassium depletion may contribute to mitochondrial dysfunction and impaired protein synthesis, although these mechanisms remain incompletely understood [16,22,23,24,25,26,27,28,29,30,33].

## 7. Principles of Diagnosis

Evaluation of potassium disorders should extend beyond measurement of serum potassium concentration. Although serum potassium remains the principal laboratory parameter used in clinical practice, it represents less than 2% of total-body potassium and therefore provides only limited information regarding intracellular potassium stores. Diagnosis requires integration of clinical findings, biochemical assessment, and evaluation of the underlying disease [7,11,13].

The initial diagnostic approach should determine whether hypokalemia results from reduced potassium intake, gastrointestinal potassium loss, renal potassium wasting, or transcellular redistribution. Clinical history should include assessment of medication use (particularly diuretics, laxatives, insulin, and β_2_-adrenergic agonists), gastrointestinal symptoms, dietary habits, endocrine disorders, and family history of inherited renal tubular diseases [7,11,13].

Laboratory evaluation should include serum potassium, magnesium, sodium, chloride, bicarbonate, creatinine, glucose, and assessment of acid–base status. Measurement of urinary potassium excretion is particularly useful for distinguishing renal from extrarenal potassium losses. Elevated urinary potassium excretion in the setting of hypokalemia suggests inappropriate renal potassium wasting, whereas low urinary potassium excretion indicates gastrointestinal losses or inadequate intake [7,11,13,18].

Assessment of plasma renin activity and aldosterone concentration is indicated in patients with resistant hypertension, unexplained hypokalemia, metabolic alkalosis, or suspected mineralocorticoid excess. Early recognition of primary aldosteronism is especially important because targeted treatment may substantially improve cardiovascular and renal outcomes [15,38].

Electrocardiography should be performed in patients with moderate or severe hypokalemia, symptoms suggestive of cardiac involvement, pre-existing cardiovascular disease, or use of medications that increase arrhythmic risk. ECG abnormalities support the diagnosis but do not reliably estimate the severity of total-body potassium depletion [11,37,42].

At present, no validated clinical biomarker accurately reflects intracellular potassium stores. Although various surrogate markers—including serum potassium concentration, erythrocyte potassium content, and functional potassium tests—have been proposed, none has been validated for direct measurement of intracellular or total-body potassium stores in routine clinical practice. Therefore, diagnosis of chronic potassium depletion relies on synthesis of clinical history, laboratory findings, acid–base status, urinary potassium excretion, associated electrolyte abnormalities, and the patient’s response to potassium replacement therapy rather than on a single direct measurement [13]. Development of reliable biomarkers of intracellular potassium status remains an important area for future clinical research.

## 8. Principles of Management and Correction of Hypokalemia

Management of potassium disorders should focus primarily on identification and correction of the underlying cause. Restoration of serum potassium concentration alone is often insufficient unless ongoing potassium losses, acid–base disturbances, endocrine abnormalities, and concomitant magnesium deficiency are simultaneously addressed [11,13].

Potassium replacement should be individualized according to the severity of hypokalemia, renal function, ongoing losses, acid–base status, magnesium status, ECG findings, route of administration, and risk of hyperkalemia [11,13,15,38].

Oral potassium supplementation is the preferred treatment for patients with mild-to-moderate hypokalemia who are clinically stable and able to tolerate enteral therapy. Potassium chloride is generally recommended because it simultaneously corrects potassium deficiency and chloride depletion, particularly in patients with metabolic alkalosis [11,13].

Intravenous potassium replacement should be reserved for patients with severe hypokalemia, life-threatening arrhythmias, profound muscle weakness, inability to tolerate oral therapy, or gastrointestinal dysfunction preventing adequate absorption. Intravenous administration requires continuous electrocardiographic monitoring and frequent reassessment of serum potassium concentrations because excessively rapid correction may result in dangerous hyperkalemia [11].

Correction of hypomagnesemia is a critical component of therapy. Magnesium deficiency impairs renal potassium conservation and frequently prevents successful correction of hypokalemia. Failure to recognize concomitant magnesium depletion is a common cause of persistent or recurrent hypokalemia despite apparently adequate potassium replacement [11,13].

Treatment should also address the underlying mechanism responsible for potassium depletion. This may include modification of diuretic therapy, treatment of gastrointestinal potassium losses, optimization of glycemic control, management of endocrine disorders such as primary aldosteronism, correction of acid–base disturbances, and treatment of chronic kidney disease when present. In patients requiring long-term diuretic therapy, potassium-sparing agents may reduce urinary potassium loss when clinically appropriate [11,13,15,38].

Dietary counseling should encourage adequate intake of potassium-rich foods in patients without contraindications such as advanced chronic kidney disease or impaired renal potassium excretion. Foods particularly rich in potassium include dried fruits, legumes, potatoes, leafy green vegetables, bananas, avocados, fish, and other minimally processed foods. Nevertheless, dietary modification should complement rather than replace treatment of the underlying cause of potassium depletion [2,13,53,57,58].

Because disturbances of potassium homeostasis frequently coexist with metabolic, cardiovascular, renal, and endocrine disorders, management is often most effective when integrated into a comprehensive strategy aimed at improving overall cardiometabolic health rather than correcting serum potassium concentration alone.

## 9. Conclusions

Disturbances of potassium homeostasis represent a heterogeneous group of disorders that extend beyond isolated hypokalemia. Differentiation between hypokalemia, total-body potassium depletion, and intracellular potassium deficiency provides a more comprehensive framework for understanding the metabolic and clinical consequences of potassium imbalance.

Current evidence indicates that chronic potassium depletion may contribute to systemic inflammation, insulin resistance, mitochondrial dysfunction, metabolic alkalosis, and anabolic resistance through multiple interacting mechanisms involving oxidative stress, altered membrane transport, neurohormonal activation, impaired cellular bioenergetics, and endocrine dysregulation. However, these associations are complex and frequently bidirectional. Potassium deficiency should therefore be regarded as an important component of integrated metabolic dysfunction rather than an isolated pathogenic factor.

Recognition of potassium disorders requires comprehensive clinical assessment because serum potassium concentration alone does not adequately reflect intracellular potassium status or total-body potassium stores. Accurate diagnosis depends on evaluation of the underlying disease, acid–base balance, renal potassium handling, concomitant electrolyte abnormalities, medication use, and associated endocrine or gastrointestinal disorders.

From a therapeutic perspective, successful management requires not only potassium replacement but also correction of the underlying cause, treatment of concomitant magnesium deficiency, optimization of acid–base status, and management of associated cardiometabolic and renal disorders. Such an integrated approach is essential for preventing recurrence and improving long-term clinical outcomes.

Future research should focus on the development of reliable biomarkers of intracellular potassium deficiency, clarification of the independent contribution of chronic potassium depletion to progression of chronic diseases, and determination of whether targeted restoration of intracellular potassium homeostasis improves long-term cardiovascular, metabolic, renal, and functional outcomes beyond normalization of serum potassium concentration alone.

## Figures and Tables

**Table 1:** Conceptual framework of disturbances of potassium homeostasis.

Condition	Definition	Typical Mechanisms	Laboratory Findings	Potential Metabolic Consequences
Hypokalemia	Decreased serum potassium concentration (<3.5 mmol/L)	Renal potassium loss, gastrointestinal loss, transcellular potassium redistribution	Decreased Serum K^+^; total-body potassium may be normal or decreased	Neuromuscular dysfunction, cardiac arrhythmias, impaired insulin secretion, altered acid–base balance
Total-body potassium depletion	Reduction of total body potassium stores irrespective of serum potassium concentration	Chronic inadequate intake, renal or gastrointestinal potassium loss, prolonged negative potassium balance	Serum K^+^ may be normal or decreased	Chronic potassium depletion, muscle weakness, impaired renal concentrating ability, metabolic disturbances
Intracellular potassium deficiency	Reduced intracellular potassium content despite variable serum potassium concentration	Impaired Na^+^/K^+^-ATPase activity, altered transcellular potassium distribution, chronic potassium depletion	No direct clinical biomarker; suspected from clinical context	Mitochondrial dysfunction, impaired glucose metabolism, anabolic resistance, altered inflammatory signaling

**Table 2:** Conceptual framework of metabolic consequences of potassium homeostasis disturbances.

Mechanism	Principal Pathways	Potential Consequences
Systemic inflammation	Oxidative stress, altered Na^+^/K^+^-ATPase activity, possible NLRP3 inflammasome activation	Chronic low-grade inflammation, further disturbance of potassium homeostasis
Insulin resistance and impaired insulin secretion	Altered β-cell electrophysiology, impaired insulin signaling, reduced glucose utilization	Glucose intolerance, impaired insulin sensitivity
Mitochondrial dysfunction	Altered mitochondrial potassium homeostasis, impaired oxidative phosphorylation, increased ROS production	Reduced ATP synthesis, fatigue, reduced exercise tolerance
Metabolic alkalosis	Increased renal H^+^ secretion, enhanced bicarbonate reabsorption, RAAS activation	Maintenance of potassium depletion, impaired tissue oxygen delivery
Anabolic resistance	Mitochondrial dysfunction, inflammation, impaired insulin signaling, reduced anabolic response and protein synthesis	Loss of muscle mass, delayed tissue repair, reduced physical performance

## Data Availability

The original contributions presented in this study are included in the article. Further inquiries can be directed to the corresponding author.
